# The Value of Contrast-Enhanced Ultrasonography Combined with Real-Time Strain Elastography in the Early Diagnosis of Prostate Cancer

**DOI:** 10.14336/AD.2017.0704

**Published:** 2018-06-01

**Authors:** Ying Chang, Jingchun Yang, Hua Hong, Huijuan Ma, Xin Cui, Li Chen

**Affiliations:** ^1^Departments of Ultrasonography, Xuan Wu Hospital, Capital Medical University, Beijing 100053, China; ^2^Departments of Ultrasonography, Inner Mongolia Autonomous Region People’s Hospital, Hohhot 010017, China; ^3^Departments of Urology, Xuan Wu Hospital, Capital Medical University, Beijing 100053, China; ^4^Departments of Pathology, Xuan Wu Hospital, Capital Medical University, Beijing 100053, China

**Keywords:** elastography, contrast agent, prostatic neoplasms, biopsy, ultrasonography

## Abstract

To evaluate the performance of a combination of real-time strain elastography (RTSE) and contrast-enhanced transrectal ultrasound (CETRUS) for prostate cancer detection. Patients with serum prostate-specific antigen (PSA) levels of ≥4.0 ng/ml were prospectively enrolled between June 2014 and December 2016. 153 prostate nodules diagnosed by conventional ultrasound were prospectively enrolled and examined by CETRUS and RTSE before a biopsy. Multivariate logistic regression models were established for CETRUS, and CETRUS combined with RTSE to diagnose prostate malignancy. The diagnostic performances of CETRUS, RTSE, and their combined use were evaluated with the receiver operating characteristic (ROC) curve. The multivariate logistic regression for CETRUS combined with RTSE showed that enhanced strength, enhanced uniformity, and elasticity scores were the independent predictors of prostate malignancy. The area under the ROC curve of CETRUS combined with RTSE (0.921±0.023) was higher than that of CETRUS and RTSE (0.88±0.029 and 0.80±0.038, respectively; both *p*<0.05). Moreover, the sensitivity, accuracy and negative predictive value of CETRUS combined with RTSE were 92.1%, 86.2%, and 84.6%, respectively. The omission diagnostic rate of CETRUS combined with RTSE (7.9%) was reduced. And the diagnostic accuracy of CETRUS combined with RTSE was significantly higher than that of CETRUS and RTSE (*p*<0.05). While the diagnostic accuracy of CETRUS was close to the RTSE, the difference was not statistically significant (*p*>0.05). The combined RTSE with CETRUS approach significantly improved the sensitivity and overall accuracy for correctly identifying prostate cancer.

Histological examination of prostatic tissues and biopsies is the only method of confirming the existence of prostate cancer. Transrectal ultrasound (TRUS)-guided sextant biopsies of the prostate is a common diagnostic procedure for prostate cancer. Guided by TRUS, spatially distributed biopsies (10 cores) are usually collected for the detection of prostate cancer. However, the sextant biopsy approach may miss up to 36% of cancers in glands smaller than 30 cm^3^ and up to 64% of cancers in larger glands [1,2]. An optimal systematic biopsy approach may require 12 to 14 cores [2,3] or even a saturation biopsy [4], but increasing the number of cores have to be weighed against increasing the risk of detecting benign diseases and the risks of the biopsy itself [2,5].

Several promising imaging techniques to identify prostate cancer lesions accurately and to provide better visual guidance for biopsy are under investigation. Real-time strain elastography (RTSE) is a novel tool for detecting cancer using the assessment of tissue elasticity based on the fact that most solid tumors differ in their consistency from the surrounding tissue because the cell density is usually greater in cancer, leading to change in tissue elasticity/stiffness [6,7]. Several groups have investigated the accuracy of RTSE for the detection of prostate cancer [8].

Contrast-enhanced transrectal ultrasound (CETRUS) involves administration of micro-bubble contrast agents to reveal critical and sensitive information on tissue perfusion and blood flow. Micro-bubble contrast agents enhance Doppler signal from regions with increased microvessel density and can improve the detection of prostate cancer [9,10]. CETRUS shows contrast medium dynamics and tumor vessels [11]. Increased and rapid contrast medium inflow, as well as increased wash-out, has been reported for cancer [12]. CETRUS enables the visualization of prostate areas with abnormal vascularity and allows for targeted biopsy, which could improve the positive rate of biopsies [10].

RTSE discriminates hard (solid tumor) from adjacent soft tissue (benign tissue), while CETRUS reveals the vascular pattern of micro-vessels. Therefore, both CETRUS and RTSE allow targeted biopsy of possibly cancerous tissues, i.e. those that are harder and/or more vascularized. At present, there are few studies about the combination of multiple ultrasound techniques for the screening of prostate cancer. Therefore, the major goal of the current investigation was to evaluate the performance of combined RTSE and CETRUS approach for prostate cancer detection. CETRUS enables the visualization of prostate areas with abnormal vascularity. Adding this information to current gray-scale and RTSE imaging methods might improve the visualization and detection of prostate cancer. We explored whether combined RTSE and CEUS approach in a multiparametric setting would improve cancer visualization before a biopsy.

## MATERIALS AND METHODS

### Study population

This study performed in Xuanwu Hospital Capital Medical University between June 2014 and December 2016. One hundred and fifty-three eligible patients were recruited; their characteristics and clinical data were summarized in Table 1. The average age of patients was 70.1±7.6 (54-75), and the mean PSA levels were 20.7±8.9 ng/ml (4.2-53.1 ng/ml). Inclusion criteria were: 1) PSA levels ≥4.0 ng/ml; 2) patients with peripheral zone lesions shown on ultrasound. Exclusion criteria were: 1) patients with severe cardiopulmonary dysfunction; 2) coagulation function disorder; 3) history of allergy to any drug or food; 4) treatment history of prostate cancer; 5) patients with clinical diagnosis prostatitis. The diagnostic accuracy of TRUS, RTSE, CETRUS, and RTSE/CETRUS for prostate cancer of patients was evaluated using the pathological examination of the biopsy as the reference standard. The Ethics Committee of Xuanwu Hospital approved the study protocol. All participants signed an informed consent.

**Table 1:** Clinical and demographic characteristics of the study population.

Characteristics	Mean ± SD
Age (years)	70.1±7.6
Prostate-specific antigen (ng/ml) Prostate volume (ml)	20.7±8.9 53.27±28.34
Pathological stage *Gleason score*	N (%)
8-10	38 (24.8%)
5-7	43 (28.1%)
4	9 (5.9%)
*Benign*	
Benign prostatic hyperplasia	27 (17.6%)
Inflammatory	24 (15.7%)
Prostatic intraepithelial neoplasia	12 (7.8%)

### Transrectal ultrasound examination

**Figure 1.**
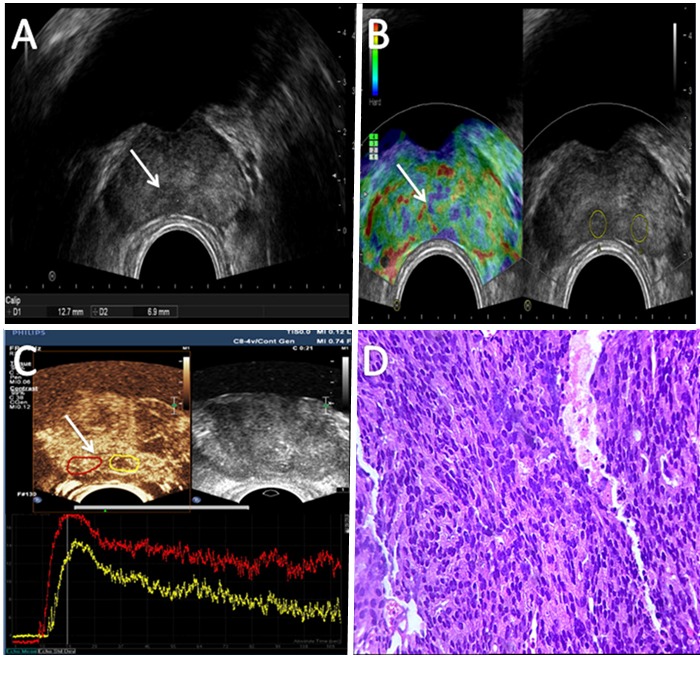
Ultrasonography of prostate cancer. (A) Transverse transrectal ultrasound (TRUS) image showing a round hypoechoic lesion in the right posterolateral prostate gland (arrow). (B) Left: transverse real-time strain elastography (RTSE) image showing a blue lesion (arrow) in the prostate base peripheral zone, with decreased elasticity. Right: grayscale ultrasound images corresponding to elastography image. (C) The upper left: transverse contrast-enhanced TRUS (CETRUS) target lesion (arrow); the upper right: grayscale ultrasound images corresponding to CETRUS image; the bottom: time intensity profiles showed the target lesion increased intensity (red curve) compared with the adjacent peripheral zone tissues (yellow curve). (D) Histopathological analysis (H&E staining) of the biopsy revealing cancer (Gleason score 6).

A digital rectal examination (DRE) was performed before the probe was placed into the rectum. Transrectal ultrasound (TRUS) was routinely performed using an HI VISION ultrasound system (Hitachi Medical, Tokyo, Japan) with a 4-9 MHz probe (EUPV53W). Each patient was scanned in the left decubitus position with buttocks positioned at the fringe of the examination bed and knees bent in the direction of the chest. On TRUS, hypoechoic lesion of the prostate marginal zone was positive (Figure 1A).

### Real-time elastography

**Figure 2.**
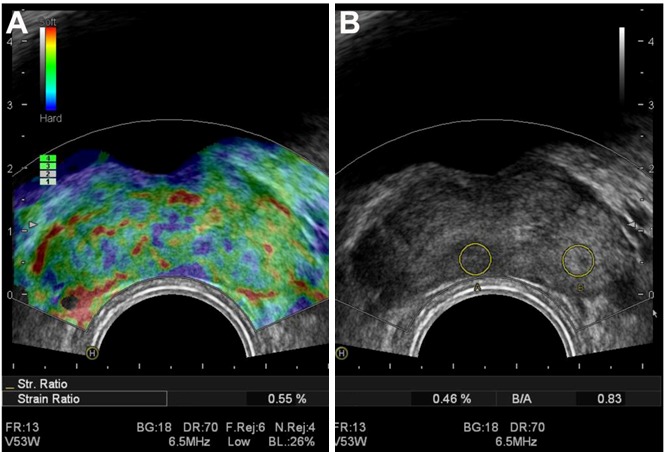
Transverse real-time strain elastography (RTSE) image of patient with prostate hyperplasia. A typical elastogram (A) and the corresponding B-mode image (B).

An HI VISION ultrasound system (Hitachi Medical, Tokyo, Japan) with a 4-9 MHz probe (EUPV53W) was employed to perform RTSE in this study. Scanning was done in the left decubitus position, as described under “Transrectal ultrasound examination” above. RTSE mode was switched on, and the elastogram was displayed on the monitor together with the grey-scale ultrasound images. To visualize tissue elasticity, different compressibility values are marked with different colors such as blue, green, and red stands for hard, medium, and soft tissues, respectively. These images were stored and transferred to a computer for further analysis (Figures 1B and 2). Images were classified into five grades according to the elastography grading standard by Kamoi *et al*. [13]. In grade I, the whole prostate was green. In grade II, lesions were green-blue, but the green area represented more than 50%. In grade III, TRUS had no hypoechoic lesions, but RTSE showed visible uneven blue area. In grade IV, the center of the lesion was green, but the periphery was blue. In grade V, the entire lesion is blue. In this study, grades I-II were considered as negative, and grades III-V as positive. The position of lesions using the anatomic landmark of prostate and surrounding tissue was recorded.

### Contrast-enhanced transrectal ultrasound examination

CETRUS was performed using an IU-Elite (Philips Healthcare, Best, The Netherlands) with an intracavitary probe with a frequency of 4-8 MHz to assess contrast-enhanced dynamics in the transverse plane of the peripheral zone only. The micro-bubble based contrast agent SonoVue (Bracco Imaging SpA, Milano, Italy) was administered intravenously at a dose of 2.4 mL and was flushed with 10 mL of saline. Then examination was started after injection of the contrast agent and was performed with a low mechanical index (MI) of 0.12. A low MI reduces micro-bubble disruption so that the contrast agent can reach the capillary bed to demonstrate microvascularity. The dynamic storage will be stored after the contrast. The QLAB quantitative software was used to analyze the characteristic of the degree curve. The following indicators were observed and recorded: (1) enhanced strength (compared with the surrounding normal glandular tissue), measured as high enhancement, equal enhancement and low enhancement; (2) enhanced uniform documented as uniform and uneven; (3) nodules as a function of time, recorded as earlier, equal to or later than the surrounding prostate parenchyma; (4) nodular peak time, measured into earlier, equal to or later than the surrounding prostate parenchyma.

Image interpretation

A different sonographer who was not aware of the CETRUS, RTSE data and pathological results analyzed the baseline grayscale images of hypoechoic tumors. Furthermore, two additional sonographers who were blinded to the grayscale imaging and pathological results independently reviewed all CETRUS and RTSE data frame by frame on the scanner. When their evaluations differed, the images were evaluated by a third experienced investigator with 5 years of extensive CETRUS and RTSE prostate experience. A consensus was reached for CETRUS and RTSE image selection discrepancies.

### Biopsy and pathological diagnosis

**Figure 3.**
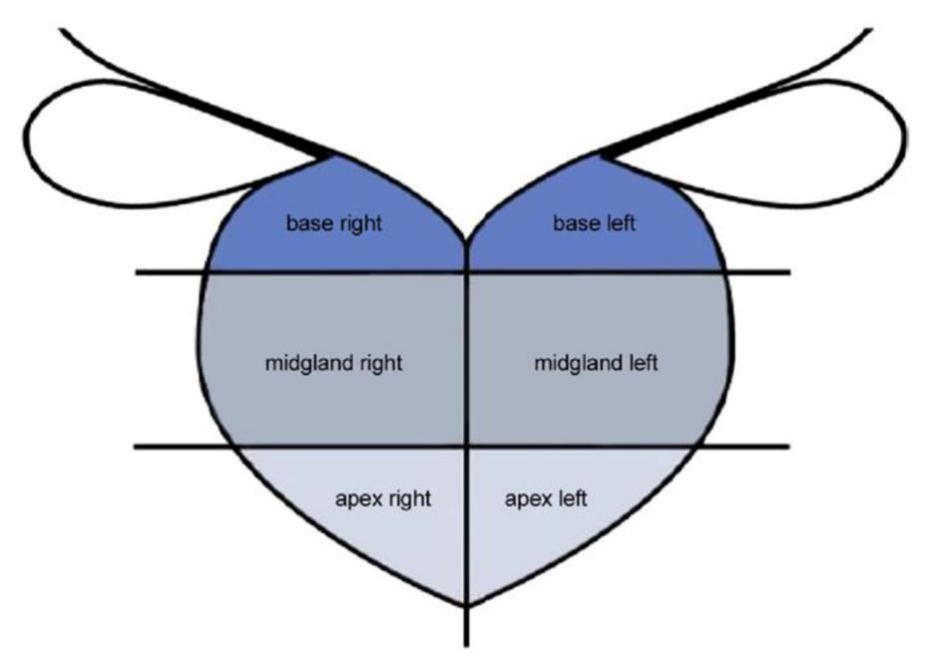
Prostate zone anatomy. Peripheral zone is divided into base, mid gland and apex at each site. Study did not focus on inner gland.

All patients underwent a TRUS-targeted biopsy of the prostate using an 18-G needle. The prostate was divided into six regions in the peripheral zone (Figure 3). Patients without positive lesion underwent 10 cores biopsies. Patients with positive lesion by the RTSE or CETRUS combination underwent 10 cores biopsies and additional two biopsies into suspicious lesions of the peripheral zone.

Pathological analysis was performed by a specialized prostate pathologist with >5 years of experience. Puncture pathological diagnosis is divided into prostate cancer and the corresponding Gleason score, benign prostatic hyperplasia, inflammation, etc.

### Statistical analysis

SPSS 18.0 (IBM, Armonk, NY, USA) software was employed for statistical analyses of data. Continuous variables were presented as a mean ± standard deviation whereas categorical variable was illustrated as frequency. Ultrasound contrast indicators of prostate nodules and the elastic score of prostate nodules were used as independent variables, with pathological results as a dependent variable along with the logistic regression equation to fit the regression model. Then, to test and draw the receive operating characteristic (ROC) curve, we calculated the area under the curve and analyzed the effectiveness of three methods of diagnosis. In every statistical analysis, a p-value of less than 0.05 was considered to indicate a significant difference.

## RESULTS

A definitive diagnosis was formulated through histopathological confirmation based on needle biopsy in all patients included in this study. The study comprised 90 patients with prostate cancers (38 with Gleason score 8-10, 43 with Gleason score 5-7, and 9 with Gleason score 4) and 63 patients with benign lesions (27 with benign prostatic hyperplasia, 24 with inflammatory changes, and 12 with prostatic intraepithelial neoplasia).

### CETRUS, RTSE and the combined diagnosis of prostate hypoechoic nodules

Ultrasound contrast parameters and elasticity scores of prostate nodules were used as independent variables. Pathological results were used as dependent variables and multivariate logistic regression analysis. The parameters were defined in Table 2. Logistic regression analysis showed that the regression equation was Logit (P) =- 4.396+3.414X1+3.794X2. CETRUS combined with RTSE; the regression equation was as follows: the regression equation is Logit (P) =-4.809+2.897X1+3.401X2+1.785X5; X1, X2, X5 represent enhanced strength, enhanced uniformity, and elasticity scores, respectively. The regression equation was statistically significant (χ2 = 18.014, 14.066, P <0.05).

**Figure 4.**
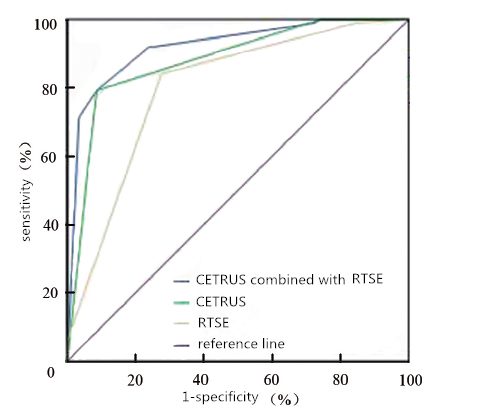
CETRUS, RTSE and the combined diagnosis of prostate nodules ROC curve.

The results of the analysis show CETRUS, RTSE and the combined diagnosis of prostate nodules by ROC curve (Fig. 4): the area under the ROC curve of the combined diagnosis (0.921±0.023) greater than the area under the ROC curve (0.88±0.029,0.80±0.038) diagnosed separately by CETRUS and RTSE.

The ROC curve showed that an elasticity score of ≥3.5, which is the diagnosis of malignant nodules at the cut-off point. CETRUS diagnosis of malignant nodules was at a predictive probability cut-off point (p-value) ≥0.65. CETRUS, RTSE combined with the diagnosis of malignant nodules had a predictive probability cut-off point (p-value) ≥0.70. The sensitivity, specificity, exactness, positive projecting estimate and negative predictive use of the above diagnostic approaches were obtained. The diagnostic accuracy of the combined diagnosis was 86.2%, which was significantly higher than that of CETRUS and RTSE respectively (83.6%, 80.0%). The diagnostic sensitivity was 92.1%, which was significantly greater than CETRUS (79.2%) and had a higher negative predictive value. Also, the group found that the rate of missed diagnosis was significantly lower than CETRUS diagnosis alone (7.9%, 20.8%), and less than RTSE diagnosis alone (15.8%).

## DISCUSSION

Based on the low sensitivity and accuracy of TRUS, various ultrasound imaging techniques (e.g., RTSE, CETRUS, and Doppler) have been introduced to optimize the visualization of prostate cancer. Indeed, sensitivity and specificity for prostate cancer detection may be enhanced by adding functional tissue information to structural tissue information [14]. The aim of this study was to evaluate the performance of a combination RTE- and CETRUS-guided biopsy for prostate cancer detection. Results showed that the RTSE/CETRUS combination had a higher sensitivity and accuracy than CETRUS alone.

**Table 2:** Ultrasonographic risk factors for maligant prostate lesions.

ultrasound indicators	variable	assignment
enhanced strength	X_1_	hyper-enhanced=0, hypo-enhanced or equal enhanced=1
enhanced uniformity	X_2_	uniformity =0, ununiformity =1
the beginning of enhancement time	X_3_	early in the surrounding organization =0 later or equal in the surrounding organization =1
the peak of enhancement time	X_4_	early in the surrounding organization =0 later or equal in the surrounding organization =1
elasticity scores	X_5_	1~3 =0, 4~5 =1
Pathological	Y	benign=0, malignant=1

TRUS-guided systematic biopsies have a low sensitivity. Grabski et al. [15] reported in a prospective multicenter study that the detection rates of prostate cancer were up to 41% by taking only six targeted cores when using a computer-based analysis of the TRUS signal. Simon et al. [16] reported prostate cancer detection rates of 45% with systematic cores. These results are akin to findings in the present study when using TRUS only.

Stark et al. [17] found that using CETRUS could improving the diagnostic rate of prostate cancer. A hypoechoic lesion on TRUS with plentiful regional flow signals is not suggestive of prostate cancer. Some previous studies [18,19] have suggested that more advanced cancers tend to display more angiogenesis. However, a clear relationship between histopathology and production of vessels has not been seen. There were seven and ten false positive CETRUS and RTSE biopsies, respectively, probably because hyperplastic tissues often appear as being hypervascularized on CETRUS [20].

Nygard et al. [21] showed that a positive RTSE was an independent marker of high-risk of prostate cancer and that RTSE-targeted biopsies improved the detection rate, but without replacing systematic biopsies. Wang et al. [22] showed that RTSE-guided biopsies improved the detection rate of prostate cancer. Nevertheless, Junker et al. [23] showed that using RTSE alone was limited by cancer size and grade, small cancers and less advanced lesions challenging to detect. Also, RTSE presents false positives that could be due to surrounding tissues that are compressed by a benign lesion, stones, calcification, or inflammatory tissues, or to small strain or shift of tissues induced by the operator.

The results of the present study are in agreement with a previous study by our group that showed that the sensitivity, specificity, and accuracy of CETRUS for the diagnosis of prostate cancer were higher than those of TRUS and power Doppler ultrasound (PDU) [10]. When compared with TRUS, the use of CETRUS and PDU avoided a considerable number of unnecessary biopsies. Nevertheless, there is still a rate of misdiagnosis with CETRUS. The combined RTSE/CETRUS approach investigated in the present study had a better sensitivity and accuracy than CETRUS alone and could reduce the rate of misdiagnosis of prostate cancer.

Also, the missed diagnosis rate was 7.9%, which was lower than CETRUS (20.8%, 15.8%, respectively). CETRUS combined with RTSE were used to diagnose benign nodules can avoid misdiagnosis.

The results of this study show that multiparametric transrectal ultrasound guided biopsy can significantly improve prostate cancer of the positive detection rate, reduce the need for unnecessary needle biopsy for the diagnosis of disease and clinical treatment to provide a more accurate objective basis. Because of the small number of cases in this study, more cases and multicenter studies are needed in later studies to confirm further that more accurate and simple imaging methods are available for early screening of prostate cancer.

However, in this study, the diagnosis of prostate cancer and histopathological grade are from the biopsy rather than the removal of pathological specimens, if the surgical resection of specimens based on the study, the results will be more reliable. Our study included only the peripheral not the central area of prostate tissue. The contrast and elasticity of the study were performed on different instruments, and therefore, the targeted puncture site was mainly determined by the anatomical landmarks. Inevitably there is a deviation between contrast and elasticity biopsy sites. Also, other approaches could also be investigated, such as RTSE combined with magnetic resonance imaging [24, 25].

In conclusion, this study analysis suggests that combining CETRUS and RTSE is feasible and does increase the diagnostic accuracy especially in the early diagnosis of prostate cancer with the targeted biopsy approach compared with CETRUS alone.
